# Conformity of modified O-ring test and maximal pinch strength for cross tape application direction

**DOI:** 10.1097/MD.0000000000010879

**Published:** 2018-06-01

**Authors:** Jung-hoon Lee, Hyun-su Choi

**Affiliations:** a Department of Physical Therapy, College of Nursing, Healthcare Sciences and Human Ecology, Dong-Eui University; b Department of Biomedical Health Science, Graduate School, Dong-Eui University, Busan, Republic of Korea.

**Keywords:** meridians, pinch strength, tape, yang, yin

## Abstract

**Background:**

Although cross tape has recently been used by clinicians for various musculoskeletal conditions, scientific studies on the direction of cross tape application are lacking.

**Methods:**

The present study aimed to investigate whether the direction of cross tape application affected the outcomes of the modified O-ring test and maximal pinch strength using a pinch gauge and the conformity between these 2 tests when cross tape was applied to the forearm muscles of individuals with no upper extremity pain and no restriction of joint range of motion.

This study used a single-blinding crossover design. The subjects comprised 39 adults (16 men and 23 women). Cross tape was applied to the dominant hand so that the 4 rows were at an angle of 45° to the right or left of the direction of the flexor digitorum superficialis muscle fibers, and then the subjects underwent a modified O-ring test and a test of maximal pinch strength using a pinch gauge. Both tests were performed in both directions, and the order of the directions and tests was randomized. SPSS 18.0 was used for statistical analysis. Cohen's kappa coefficient was used to analyze the conformity of the results from the 2 tests. The statistical significance level was *P* < .05.

**Results:**

A positive response in the modified O-ring test and maximal pinch strength were both affected by cross tape direction. The modified O-ring test and maximal pinch strength using pinch gauge results were in agreement (*P* < .00), and the kappa coefficient was significant at 1.00.

**Conclusion:**

The direction of cross tape application that produced a positive response in the modified O-ring test also produced greater maximal pinch strength. Thus, we propose that when applying cross tape to muscles, the direction of the 4 lines of the cross tape should be 45° relative to the direction of the muscle fibers, toward the side that produces a positive response in the modified O-ring test or produces the greatest maximal pinch strength using a pinch gauge.

## 1 Introduction

Spiral taping is a method developed by the acupuncturist Nobutaka Tanaka in the 1980s. It can be used to treat various musculoskeletal and internal medical disorders using the electromagnetic current in the muscles and skin and applying cross tape to acupuncture points or reaction points in tense areas.^[1,2]^ In previous studies, applying cross tape to the ankles and neck improved proprioception and balance,^[3]^ and application of cross tape before menstruation was reported to alleviate menstrual pain.^[4]^ It has also been used to alleviate the low back pain.^[5,6]^ Cross tape application to the ankle improved balance in healthy individuals,^[7]^ whereas application to the foot improved balance in stroke patients.^[2]^ Meanwhile, application of cross tape to trigger points of the upper trapezius muscle alleviated pain but did not effectively reduce muscle tone.^[8]^

Cross tape has a rectangular shape, consisting of a lattice of 4 lines of tape in 1 direction and 3 lines of tape in the orthogonal direction,^[1,9]^ which, as odd and even numbers, respectively, represent *yin* and *yang* in Eastern medicine.^[10]^ When applying cross tape to muscles, Tanaka stated that the cross tape should first be placed with the 4 lines oriented 45° to the left or right of the direction of muscle fibers, whichever direction produced a positive response in the modified O-ring test (stronger finger flexion).^[1]^ Tanaka broadly classified the direction of flow in the meridian system into 2 categories: the first is the left direction type, in which the *yin* meridians flow from the right lower limb to the left upper limb at the front of the body and the *yang* meridians flow from the left upper limb to the right lower limb at the back of the body, and second is the right direction type, in which the *yin* meridians flow from the left lower limb to the right upper limb at the front of the body and the *yang* meridians flow from the right upper limb to the right lower limb at the back of the body.^[1]^ When cross tape is applied to relieve muscle tension and alleviate pain, it was reported that the 4 lines of the cross tape should be oriented 45° left or right of the direction of the muscle fibers, choosing the side that elicits a positive response in the modified O-ring test.^[1]^

However, even though cross tape is currently used by clinicians in several countries for various musculoskeletal disorders, there has only been theoretical discussion of the direction of cross tape application, with no scientific studies. The aim of our study was to examine whether the direction of cross tape application affected the results of the modified O-ring test and maximal pinch strength measured using a pinch gauge and to investigate the conformity between these 2 tests when cross tape was applied to the forearm muscles of individuals with no upper extremity pain or restriction of joint range of motion (ROM).

## 2 Method

### 2.1 Participants

Using G-Power 3.1 (University of Dusseldorf, Dusseldorf, Germany) with an alpha level of 0.05, power of 0.8, and effect size of 0.4, the required sample size was calculated to be 34 subjects. However, to account for dropout, we recruited 40 male or female adults with no upper extremity pain and no restriction of joint ROM. The exclusion criteria were pain or restricted joint ROM in the neck and upper extremity due to orthopedic or neurological injury in the last 3 months, history of medication to treat musculoskeletal disease within the last 3 months, no sensation or with a tingling sensation in the upper extremity, and/or development of contact dermatitis after application of cross tape. After excluding 1 subject during screening (wrist pain), a total of 39 subjects participated in the study. All participants provided written informed consent, and this study was approved by the institutional review board of Dong-eui University. The general characteristics of the participants are shown in Table 1.

**Table 1:**
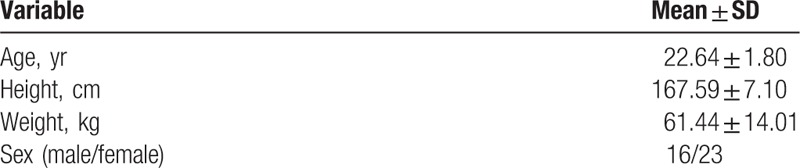
General characteristics of the subjects (n = 39).

### 2.2 Procedures

**Figure 1:**
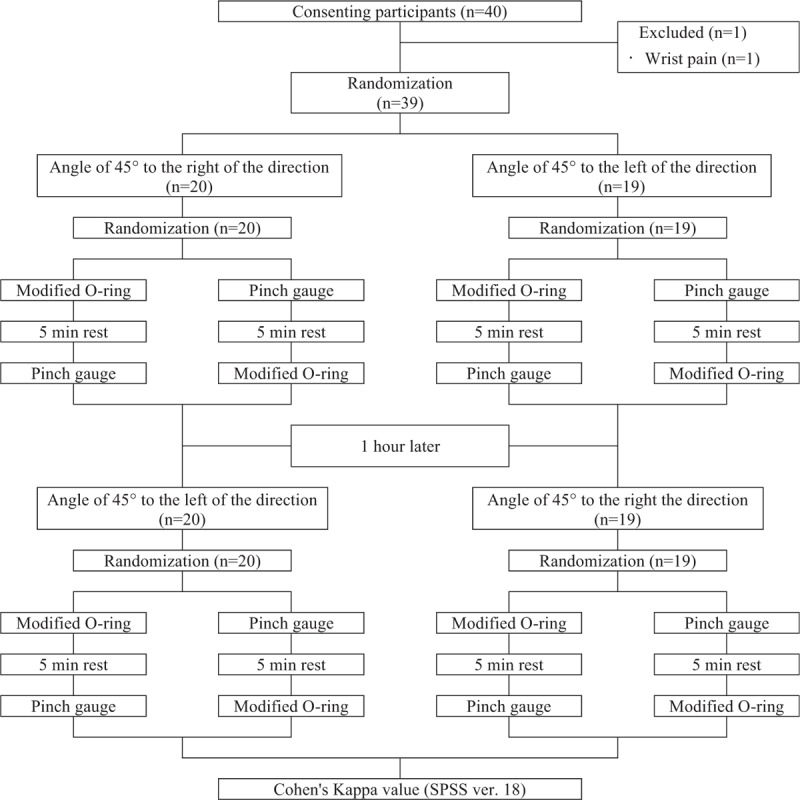
Study flowchart.

This study used a single-blinding crossover design. Cross tape was applied so that the 4 rows were at an angle of 45° to the right or left, relative to the direction of the fibers of the flexor digitorum superficialis muscle on the dominant hand, and then the subjects underwent a modified O-ring test and a test of maximal pinch strength using a pinch gauge. Both tests were performed for both directions, and the order of the directions and tests was randomized. To prevent muscle fatigue, the subject rested for 5 minutes between each measurement. A flowchart of the study is shown in Figure 1.

### 2.3 Intervention

**Figure 2:**
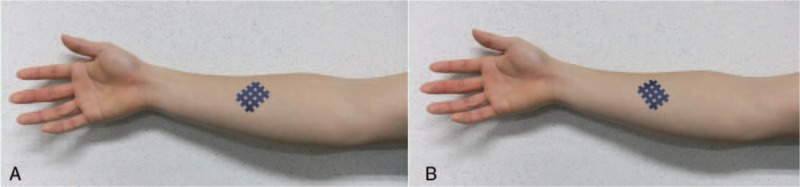
Cross tape application.

The cross tape (BB Tape; Wetape Inc, Paju, Korea) was made of polyester coated with acrylic adhesive and formed a rectangular lattice of 3 and 4 lines of tape intersecting at evenly spaced intervals. The subjects were blindfolded to blind them to the direction of cross tape application. Subjects were seated upright in a chair, the shoulder joint was moved so that the arm was next to the body, the elbow joint was flexed to 90°, and the forearm and wrist were placed in a neutral position. The cross tape was then applied with the 4 lines of tape oriented 45° to the left (Fig. 2A) or right (Fig. 2B) of the direction of the flexor digitorum superficialis muscle fibers; the order of the directions was randomized. Since the meridian spreads with the velocity of a light wave,^[11,12]^ finger flexion strength was immediately measured using maximal pinch strength and the modified O-ring test.

### 2.4 Measurements

#### 2.4.1 Maximal pinch strength

**Figure 3:**
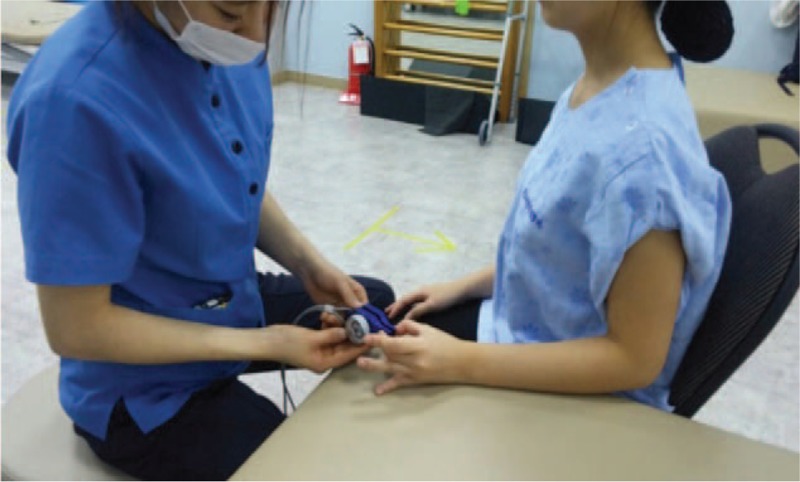
Maximal pinch strength using pinch gauge.

A portable pinch gauge (Baseline, Fabrication Enterprises Inc, Irvington, NY) was used to measure differences in maximal pinch strength depending on the direction of cross tape application. In accordance with the American Society for Hand Therapists guidelines,^[13]^ subjects sat upright and blindfolded, the shoulder joint of the dominant hand was adducted, the elbow was flexed 90°, and the forearm and wrist were placed in a neutral position. To maintain an angle of 90° at the angle joint and prevent compensatory action during measurement of maximal pinch strength, subjects placed their forearm on a table with adjustable height (Fig. 3). After applying cross tape with the 4 lines of tape oriented 45° right or left of the direction of the flexor digitorum superficialis muscle fibers, the subject grasped a pinch gauge with the thumb and middle finger and maintained pain-free maximal isometric contraction for 3 seconds; this was repeated 3 times at 1-minute intervals.^[14]^ The mean of the 3 measurements was used in the analysis.^[14]^

#### 2.4.2 Modified O-ring test

The bi-digital O-ring test was developed by Omura in the 1970s as a noninvasive, sensitive method to obtain information about various pathologies. The subject forms an O-ring shape with the thumb and a selected finger, and the examiner assesses the resistance of the finger flexors while trying to forcefully open the O-ring.^[15]^ Tanaka used the modified O-ring test, with the O-ring formed by the thumb and middle finger, to determine the appropriate direction of cross tape application.^[1]^

**Figure 4:**
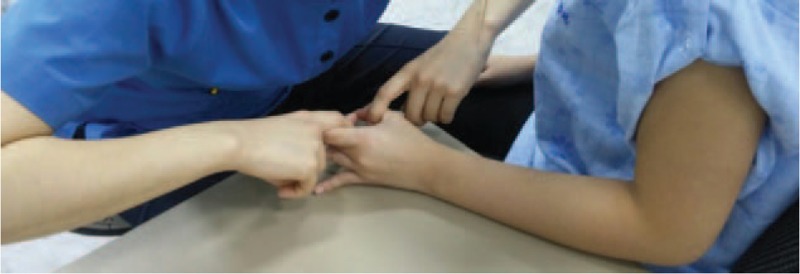
Modified O-ring test.

During the modified O-ring test, to ensure a constant force to open the O-ring, the test was performed by an examiner with an intrarater reliability of at least 0.8 using a tension biofeedback device (4D-MT, RELIVE, Gimhae, Korea). Measurements were taken in the same position as maximal pinch strength; subjects were blindfolded and seated upright; the shoulder joint of the dominant hand was adducted so that the arm was alongside the body; the elbow joint was flexed 90°; and the forearm and wrist were placed in a neutral position (Fig. 4). After applying cross tape with the 4 lines of tape oriented 45° right or left of the direction of the flexor digitorum superficialis muscle fibers, the subject made an O-ring shape with their thumb and middle finger, and the examiner applied a constant force to try to open the O-ring.^[1]^ To maintain the elbow joint angle at 90° and prevent compensatory action during measurement of the modified O-ring test, the subject placed their forearm on a table with adjustable height.

### 2.5 Statistical analysis

SPSS 18.0 (IBM Corp, Armonk, NY) was used for statistical analysis of the measurements. Cohen's kappa coefficient was used to analyze the conformity between the 2 measurements, with a statistical significance level of *P* < .05. To grade the kappa coefficient, we used the interpretation of Landis and Koch, in which a value of 0–0.2 represents a slight agreement and ≥0.8 represents almost perfect agreement.^[16]^

## 3 Result

The modified O-ring test and maximal pinch strength using pinch gauge results were in agreement (*P* < .00), and the kappa coefficient was significant at 1.00 (Table 2). When the 4 lines of the cross tape were oriented 45° left of the direction of the flexor digitorum superficialis muscle fibers, 27 subjects showed a positive response in the modified O-ring test, and they also showed greater maximal pinch strength for this cross tape direction when measured using a pinch gauge (Table 2). When the 4 lines of the cross tape were oriented 45° right of the direction of the flexor digitorum superficialis muscle fibers, 12 subjects showed a positive response in the modified O-ring test, and they also showed greater maximal pinch strength for this cross tape direction when measured using a pinch gauge (Table 2).

**Table 2:**
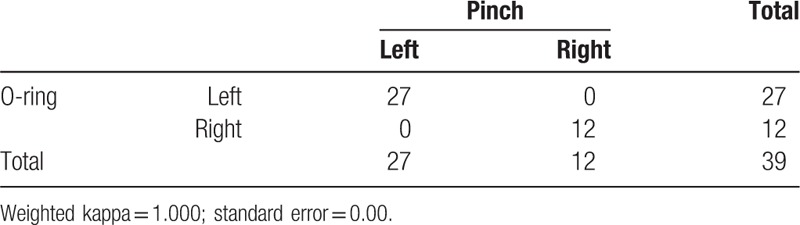
Agreement between the O-ring test and pinch gauge with cross tape.

## 4 Discussion

The present study showed that even when applying the cross tape on the same point, the direction of the cross tape application affected the outcomes of both the modified O-ring test and maximal pinch strength using a pinch gauge. Moreover, Cohen kappa coefficient showed perfect agreement between the modified O-ring test and maximal grip strength. For those subjects who showed a positive response in the modified O-ring test after having cross tape with the 4 lines 45° left of the direction of the muscle fibers, the maximal pinch strength was also greater when the cross tape was applied in the same orientation.

In the present study, we found that a positive response to the modified O-ring test was affected by the direction of cross tape application, but we found that maximal pinch strength using a pinch gauge was also affected by the direction of cross tape application. The cross tape direction that produced a positive response in the modified O-ring test also produced greater maximal pinch strength, with these results showing perfect conformity.

There is a lack of the most robust types of research (meta-analysis, systematic review, or randomized controlled trial) for the use of cross tape in treating myofascial trigger points.^[8]^ As a result, clinicians depend on the manufacturers’ Web sites for most information about cross tape.^[17]^ Cross tape is applied to acupuncture points, myofascial trigger points, tense muscles, areas of localized pain, painful joints, painful scars, and areas of headache with no consideration of directionality.^[8]^ However, cross tape does not work by simple application to painful areas; it has unique sites of application for each muscle, to maintain the overall balance of muscle tension according to the laws of tension for various different muscles.^[1]^ In addition, the direction of cross tape application needs to be varied depending on the type of meridian flow (left or right direction type).^[1]^

The direction of cross tape application can be considered in terms of the Eastern medical theory. Two types of energy with electromagnetic characteristics, *yin* and *yang*, co-exist in the body^[18]^; here, *yin* refers to a depressed state and *yang* refers to an elevated state.^[2]^ In accordance with the Eastern medical theory of enriching *yin* and draining *yang*, the foundation of Eastern medical treatment is decreasing *yang* and increasing *yin* to normal levels.^[2]^ Likewise, the aim of cross taping is to address the *yin*-*yang* balance, and thus, an excess of *yang* is given *yin* treatment and vice versa^[5,7]^; in the left direction type, the direction 45° to the right is the *yin* direction and the direction 45° to the left is the *yang* direction.^[19]^ Therefore, when the 4 lines of tape, which, as an even number, represent the *yin* meridian in Easter medicine,^[10]^ are oriented 45° to the left (the *yang* direction), this redresses the *yin*-*yang* balance. In the right direction type, the direction 45° to the left is the *yin* direction and the direction 45° to the right is the *yang* direction.^[19]^ Therefore, the *yin*-*yang* balance is redressed by orienting the 4 lines of tape, representing the *yin* meridian,^[10]^ 45° to the right (the *yang* direction). Thus, it is thought that when the 4 lines of the cross tape were applied in the direction that balances the *yin* and *yang* in the electromagnetic current across the patient's muscles and skin, this increased the resistance of the finger flexors used in the modified O-ring test and the maximal pinch strength test.

Recently, one study reported no change in muscle tone after cross tape application at the upper trapezius trigger points. However, that study included no prior tests regarding cross tape direction and applied the cross tape without considering orientation.^[8]^ Another study, in which cross tape showed no significant effect in primary dysmenorrhea with a control group receiving cross tape over the greater trochanter area, but that study also showed no prior examinations or considerations about cross tape directionality.^[20]^

Although our study did not demonstrate the physiological mechanisms of pain relief and muscle relaxation due to cross tape application, it will be important in future clinical application of cross tape to perform prior tests of cross tape direction and to examine the outcomes of cross tape application in the direction indicated by those tests.

Our study had some limitations. First, we did not test for the effect of cross tape direction on the modified O-ring test and maximal pinch strength using a pinch gauge in subjects with actual pain. Second, we did not test whether the cross tape direction affected pain or muscle tension in subjects with actual pain. In the future, it will be important to perform scientific studies to determine how cross tape direction affects pain in subjects experiencing pain.

## 5 Conclusion

Based on the results of our study, when applying cross tape, we propose that the 4 lines of the cross tape should be oriented at an angle of 45° to the direction of the muscle fibers toward the side that produces a positive response in the modified O-ring test or that produces a greater maximal pinch strength using a pinch gauge.

## Author contributions

**Conceptualization:** Jung-hoon Lee.

**Data curation:** Hyun-su Choi.

**Investigation:** Jung-hoon Lee, Hyun-su Choi.

**Methodology:** Jung-hoon Lee.

**Project administration:** Jung-hoon Lee.

**Supervision:** Jung-hoon Lee.

**Validation:** Jung-hoon Lee.

**Writing – original draft:** Jung-hoon Lee, Hyun-su Choi.

**Writing – review and editing:** Jung-hoon Lee.
